# Supplementary material for: Plant–atmosphere and soil–atmosphere temperature differences and their impact on grain yield of super hybrid rice under different irrigation conditions
Source: PLoS One. 2020 Dec 17;15(12):e0243580. doi: 10.1371/journal.pone.0243580 (PMC7746292; doi:10.1371/journal.pone.0243580)
Supplement: Attachment — Submitted filename: Response to Reviewers.doc [file pone.0243580.s003.doc]

Response to Reviewers

Additional Editor Comments (if provided):

1. Please correct the unit of the grain yield results, which are often written as g m-1 instead of g m-2 (i have seen many instances in the text, in Table 2 also.

The error has been revised.
